# Review of the Proteomics and Metabolic Properties of *Corynebacterium glutamicum*

**DOI:** 10.3390/microorganisms12081681

**Published:** 2024-08-15

**Authors:** Juhwan Park, Sooa Lim

**Affiliations:** Department of Pharmaceutical Engineering, Hoseo University, Asan-si 31499, Chungnam, Republic of Korea

**Keywords:** *Corynebacterium glutamicum*, proteome, central carbon metabolism, industrial microorganisms, microbial biology

## Abstract

*Corynebacterium glutamicum* (*C. glutamicum*) has become industrially important in producing glutamic acid and lysine since its discovery and has been the subject of proteomics and central carbon metabolism studies. The proteome changes depending on environmental conditions, nutrient availability, and stressors. Post-translational modification (PTMs), such as phosphorylation, methylation, and glycosylation, alter the function and activity of proteins, allowing them to respond quickly to environmental changes. Proteomics techniques, such as mass spectrometry and two-dimensional gel electrophoresis, have enabled the study of proteomes, identification of proteins, and quantification of the expression levels. Understanding proteomes and central carbon metabolism in microorganisms provides insight into their physiology, ecology, and biotechnological applications, such as biofuels, pharmaceuticals, and industrial enzyme production. Several attempts have been made to create efficient production strains to increase productivity in several research fields, such as genomics and proteomics. In addition to amino acids, *C. glutamicum* is used to produce vitamins, nucleotides, organic acids, and alcohols, expanding its industrial applications. Considerable information has been accumulated, but recent research has focused on proteomes and central carbon metabolism. The development of genetic engineering technologies, such as CRISPR-Cas9, has improved production efficiency by allowing precise manipulation of the metabolic pathways of *C. glutamicum*. In addition, methods for designing new metabolic pathways and developing customized strains using synthetic biology technology are gradually expanding. This review is expected to enhance the understanding of *C. glutamicum* and its industrial potential and help researchers identify research topics and design studies.

## 1. Introduction

*Corynebacterium glutamicum* (*C. glutamicum*), described by Shukuo Kinoshita and co-workers in Japan and called *Micrococcus glutamicus nov.* sp., was first isolated in 1957 [1,2]. The species was renamed *C. glutamicum* based on taxonomic studies and the classification of 208 species of microorganisms that produce glutamic acid [3]. In the 1960s, the commercialization of *C. glutamicum* was achieved, and early studies focused on increasing yields by understanding the pathways involved in glutamic acid production and optimizing fermentation conditions [4]. Various studies were conducted on its industrial use to produce monosodium glutamate [5]. For example, studies were performed on NH_4_^+^ uptake, the nitrogen source preferred by most microorganisms, and the mapping and identification of its proteins by two-dimensional electrophoresis [6,7]. In addition, studies were conducted on its use in osmosensors and osmoregulators for quantifying anaplerosis flux in vivo [8,9,10]. In the 1970s, significant progress was made in lysine production by exploring the potential of *C. glutamicum* to produce other amino acids [11,12]. This was followed by the development of genetic manipulation tools in the 1980s, which further increased amino acid yields and provided insights into the central carbon metabolism, leading to a better understanding of metabolic pathways [13,14,15,16].

*C. glutamicum* strain development has been used in proteome and central carbon metabolism research to produce useful substances, such as L-glutamate, L-lysine, and bioplastic [17,18,19]. Indeed, *C. glutamicum* has been subjected to continuous research and development since it was first identified and is now used industrially in various fields. Developmental efforts continue because of the unique characteristics of *C. glutamicum* [6,20,21,22,23,24]. The bacterium is expected to become a useful industrial source of raw materials for novel biotechnologies. For example, it can be used to develop strains that produce substances required in limited quantities cost-effectively, such as biosensors for pollutant detection [25,26,27,28,29,30,31,32,33,34,35]. From the 1990s to the modern era, studies on the central carbon metabolism have expanded biological understanding and industrial applications, laid the foundation for genetic and metabolic engineering, and developed more pro-friendly industrial processes [36,37,38,39]. This review provides an overview of the research trends, the industrial and environmental uses of *C. glutamicum*, and information on its metabolic processes for different central carbon sources. This review will assist researchers across various fields in producing the desired metabolites and controlling the functions of microorganisms.

## 2. *Corynebacterium glutamicum* Research Timeline and Overview

Between 2000 and 2010, a protocol for the systematic analysis of extracts from various biological sources using HPIC (high-performance ion-exchange chromatography) was introduced. In particular, protocols were developed to quantify the most important intermediates of central carbon metabolism [40], followed by the project to elucidate the complete genome sequence [38,41]. At the same time, proteome studies using N-terminal sequencing, matrix-assisted laser desorption/ionization-time of flight–mass spectrometry (MALDI-TOF-MS), electrospray ionization-mass spectrometry (ESI-MS), and phospho-proteomic analysis and membrane proteome studies were conducted [42,43,44]. Subsequently, studies revealed lactate utilization in *C. glutamicum* induced by a temperature-triggered operon during glutamate production [21] and citrate utilization in *C. glutamicum* by transcriptome and proteome analysis [22]. Several attempts have been made to identify the physiological properties of cell factories and to study physiological adaptations to non-traditional carbon sources such as benzoic acid [20,45]. Increased knowledge of systems-level characterization based on genomics, transcriptomics, proteomics, metabolomics, and flux analysis has facilitated improvements in *C. glutamicum* strains [46,47]. Studies from a proteomic perspective include analyses of proteases, membranes, and cytoplasm [48]. Interestingly, studies on proteins identified several that can indicate environmental contamination because of their responses to heavy metal exposure [49]. Furthermore, a study on the physiological adaptation of *C. glutamicum* to other carbon resources or conditions, such as benzoic acid, salt stress, and ammonium limitations, revealed global adaptation membrane responses [45,50].

The advent of the 2010s was marked by a 1.75-fold increase in the number of papers published on *C. glutamicum*. Between 2010 and 2020, focus was placed on production using *C. glutamicum*, particularly metabolic engineering, genomics, and proteomics [51,52,53,54,55]. Clarification of the regulation of biosynthesis and the metabolism of certain amino acids and improved understanding of adaptation to specific stresses and advances in genetic engineering led to efforts to develop or improve industrially applicable strains [56,57,58,59,60,61,62,63]. Furthermore, improved genetic manipulation techniques and metabolic studies provided new research tools such as CRISPR genetic toolkits, synthetic biology tools, and NGS [64,65]. For example, genetic engineering studies have suggested using *C. glutamicum* to biosynthesize chondroitin and hyaluronic acid and complex regulatory interactions related to its central metabolism [61,66,67]. In terms of metabolic engineering with the discovery of the metabolic pathways, applications related to the production of L-lysine, L-threonine, L-valine, lactic acid, succinic acid, and violacein, as well as approaches for overproduction, have been explored [51,52,60,68,69,70,71,72,73,74,75,76]. Methods of biosynthesizing aromatic compounds and natural products have also been investigated [77,78]. Interestingly, studies have also been conducted on the use of *C. glutamicum* as a multipurpose cell factory for bio-molecule production [35,53,79,80], its growth characteristics under different conditions, proteome turnover and protein secretion [81,82], and synthetic biological approaches to enable the use of different carbon sources [83,84]. The above studies confirmed that *C. glutamicum* is a microorganism of industrial importance [85].

**Figure 1:**
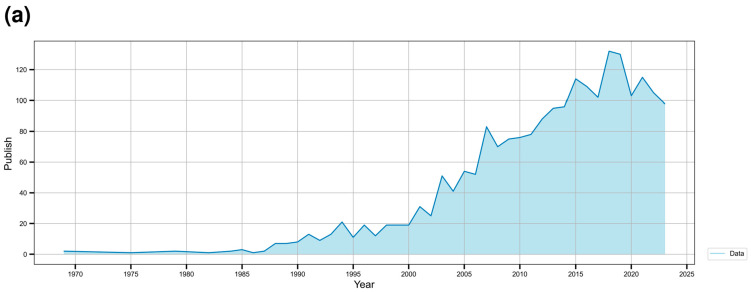

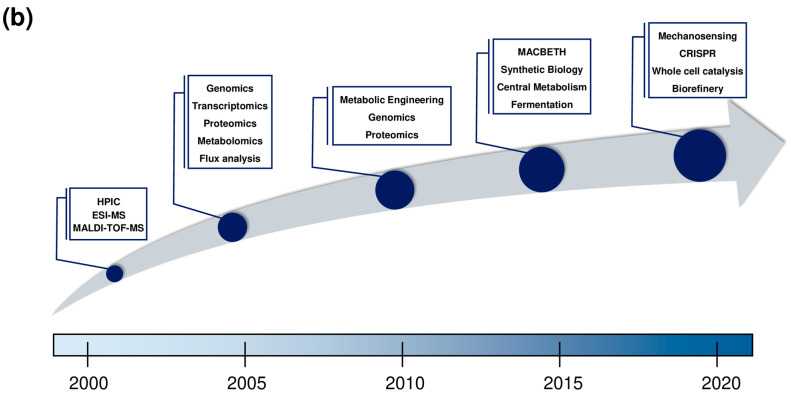
(**a**) Number of papers registered with PubMed, and (**b**) key development timeline of *Corynebacterium glutamicum* research.

**Figure 2:**
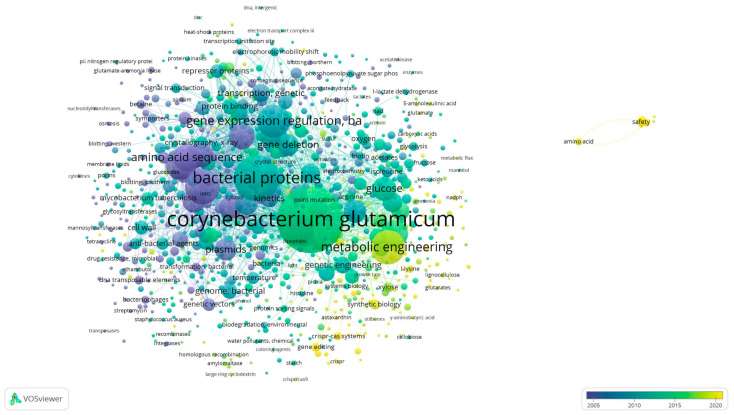
Analysis of *Corynebacterium glutamicum* research and development trends using VOSviewer.

**Figure 3:**
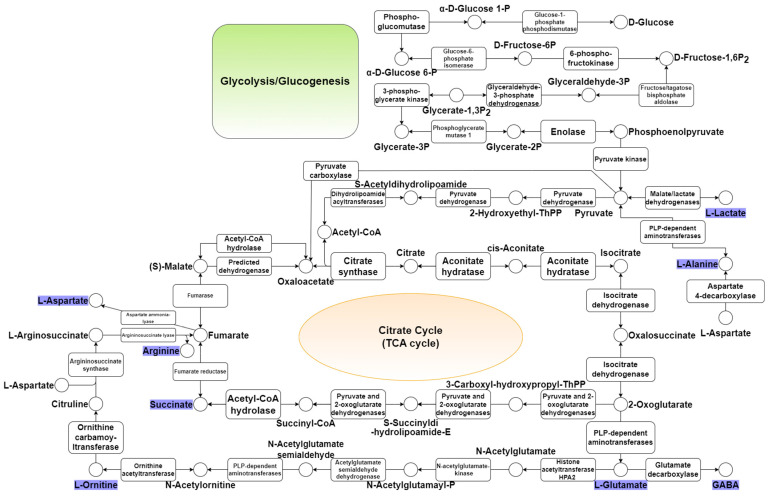
Biosynthesis pathway in central carbon metabolism.

Efforts to achieve economic material production continued in the 2020s. For example, studies successfully produced (3R)-acetoin, L-valine, L-glutamate, and branched-chain amino acids [86,87,88]. In addition, *C. glutamicum* could be used to produce mixed acids from sugars and aromatic compounds present in the lignocellulosic biomass and to process highly resistant aromatic molecules commonly present in lignocellulosic hydrolysates, such as benzoic acid, cinnamic acid, vanillic acid, and p-coumaric acid. On the other hand, lignocellulose cannot provide an accessible carbon source for *C. glutamicum* despite all genetic engineering manipulations without pretreatment and subsequent enzymatic hydrolysis of the substrate. Lignocellulose biomass must be pretreated with dilute acid to accelerate the availability of the enzyme and subsequent hydrolysis of polysaccharides [70,89,90]. *C. glutamicum* also presents the possibility of the microbial synthesis of plant flavonoids. For example, it has been used to produce eriodictyol, demonstrating drug and food development potential as a GRAS strain [91]. Furthermore, a metabolic engineering approach was used to produce compounds, such as itaconic acid, from alternative substrates (e.g., acetate) rather than sugar from *C. glutamicum* [34]. Figure 1a presents a graph depicting the number of *C. glutamicum*-related publications indexed in PubMed up to 2023 over time. Figure 1b presents the key development timeline of *C. glutamicum* research. Moreover, the mechanosensitive channel *C. glutamicum* is a specialized carrier that secretes L-glutamate into the surrounding environment rather than acting as an osmotic safety valve, which provides evidence that *C. glutamicum* originated as a gut bacterium in an avian host [92]. *C. glutamicum* is also being investigated as a potential environmental bioremediation tool [39]. Furthermore, *C. glutamicum*, a facultative anaerobic bacterium, has been suggested to be a 21st-century microbial cell of interest to carbon-free societies because *C. glutamicum* grows under aerobic conditions and produces amino acids and can be used to generate several value-added compounds, including organic acids, through metabolic migration under anaerobic conditions, [23]. Figure 2 presents a visualization of keywords associated with *C. glutamicum* using VOSviewer and Figure 3 summarizes the basic metabolic mechanisms by showing biosynthetic pathways in central carbon metabolism.

## 3. Importance of Proteome Research and Its Impact on Future Research

### 3.1. Use of C. glutamicum for the Mass Production of Useful Proteins

Research on the proteome of *C. glutamicum* has been conducted since the 2010s. This review outlines the importance of this research and its future impact. Proteome research has three main aspects. First, it might provide economically viable, mass-produced proteins for biotechnology, pharmaceutical engineering, and industry [25,28,32,93,94,95]. Furthermore, the strategic manipulation of genetic elements would enable the engineering of microorganisms tailored to produce targeted proteins. Sophisticated data analysis algorithms and high-resolution mass spectrometry enable protein synthesis and degradation (turnover) rates to be determined using stable isotope-labeled amino acids, which would allow the advantages and disadvantages of different approaches, such as 2D electrophoresis and chromatography-based analysis, to be assessed [81]. *C. glutamicum* has several advantages over *E. coli*. Accordingly, it has been considered a promising alternative prokaryotic host for recombinant protein expression. *E. coli* lacks an effective secretion mechanism. As a result, a significant portion of the proteins expressed by *E. coli* are insoluble, making product recovery difficult without the formation of inclusion bodies. Indeed, 80% of non-membrane proteins in *E. coli* are unsuitable for structural studies because of their low solubilities. More than 90% of potential pharmaceutical protein developments are terminated in the early clinical stages for the same reason [54,96,97]. In addition, therapeutic protein development is hampered by bacterial endotoxins produced by *E. coli* [98]. Bacterial endotoxins are highly toxic and can cause severe inflammatory reactions and septic shock, making them dangerous in laboratory and clinical applications [99,100]. Lipopolysaccharides (LPS) can trigger a strong immune response in humans. Therefore, it is essential to remove these contaminants from all therapeutic proteins produced using *E. coli* [101,102]. By comparison, *C. glutamicum* is a promising alternative that produces therapeutic proteins with fewer endotoxin-related problems. With *E. coli*, a complex and expensive endotoxin removal process is required, but these steps are not required with *C. glutamicum*, making the entire production process more straightforward and more economical [36,103,104]. Thus, *C. glutamicum* is considered an attractive alternative to avoid endotoxin problems as a host for therapeutic protein production while maintaining high productivity. Studies have been conducted on promoters, plasmid vector optimization, and protein secretion pathways to realize the potential of *C. glutamicum* [105]. Furthermore, the bacterium has been proven suitable for expression library construction using tools developed to enable the introduction of native, mutated, heterologous, or synthetic genes expressed by promoters [106]. Table 1 compares *C. glutamicum* and *E. coli* based on their respective characteristics. In 2018, an automated system was constructed and integrated with a robotic system named MACBETH to generate thousands of manipulated strains per month. Constructing at least 600 plasmids per day is theoretically possible using this system. Therefore, when combined with multiplex genome editing, this system could target 9,000 genes per month, making it an advanced tool for *C. glutamicum* engineering [107,108].

### 3.2. Use of C. glutamicum as a Biosensor and Diagnostic Tool

**Figure 4:**
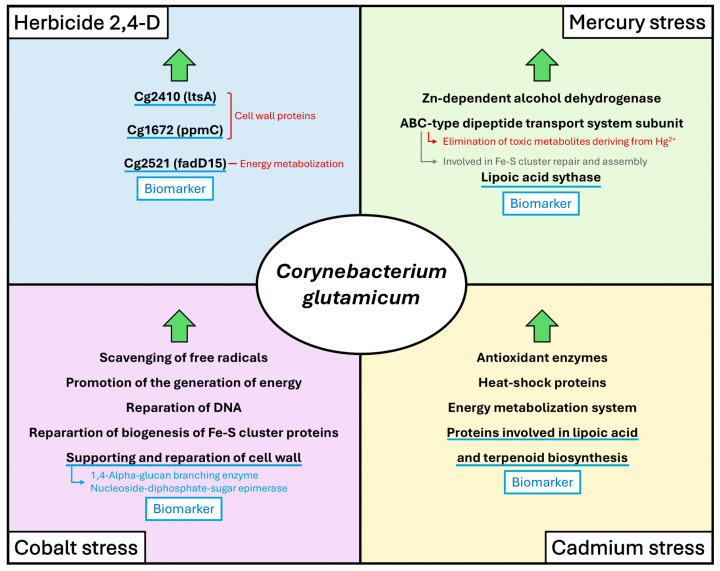
Protein expression and physiological changes in *Corynebacterium glutamicum* under various stresses: Identification of potential biomarkers.

The second main aspect of proteome research concerns using *C. glutamicum* as a biosensor or tool to diagnose exposure to hazardous substances. Studies have shown that *C. glutamicum* can be used to confirm changes in protein expression by exposure to herbicides, cobalt, silver, cadmium, or mercury. These studies could greatly aid the understanding of the toxic mechanisms of metals and how *C. glutamicum* adapts to heavy metal stress. The protein spots that are up- or down-regulated by toxic mechanisms have been studied, and the defense mechanisms of *C. glutamicum* against heavy metal stress have been identified. Among these defense mechanisms, the induction of stress promoters responding to heavy metals has been suggested to be the first indicator of pollutants. Moreover, detecting such responses has been reported to be an alarm signal with potential use as a biomarker for detecting the presence of heavy metals in living organisms [49,109,110]. In addition, genome manipulation techniques, ranging from traditional allele exchange-based recombination methods to base editors, have made significant advances and now have potential use for diagnosing exposure to more diverse substances [111]. Other researchers have shown that a combination of fluorescence-activated cell sorting (FACS) and proteomics is suitable for the selection and molecular characterization of microbial cells containing different concentrations of metabolites [112]. Furthermore, *C. glutamicum* could be used to analyze mutant libraries for cells with increases or decreases in cAMP levels as cAMP biosensors during FACS analysis [113]. *C. glutamicum* could also be used as a biosensor to identify high-yield strains and observe product accumulation in real-time by quantitatively monitoring intracellular metabolites in vivo [114]. Figure 4 presents the proteins expressed and the physiological changes observed under various stresses, highlighting the proteins that could be used as biomarkers.

### 3.3. Biological Purification

The third main aspect of proteome research concerns the multi-omics understanding of *C. glutamicum* in the context of pollutant bioremediation [115]. Large-scale industrial production has been limited by environmental pollution and high production costs. *C. glutamicum* can optimize L-lysine production at 33 °C, and adaptive evolution is likely to minimize the use of cooling water during the fermentation process. In 2021, a strain was developed that could produce 41.00 g/L of L-lysine in 60 h by batch fermentation that did not require sterilization [116]. Nevertheless, the continuous fermentation performance of the system was less than ideal, and improvements were needed to reduce pollution. Despite the various problems, bio-production industries must realize green production by developing cost-effective processes, lowering environmental burdens, and minimizing resource wastage [117].

### 3.4. Association between Central Carbon Metabolism and Proteomics

The connection between central carbon metabolism and proteomics is important in microbiology and biotechnology research. These two fields are closely connected, and each study deepens the understanding of the other.

By examining the expression level, modification, and interaction of enzymes acting in the pathway, such as the tricarboxylic acid (TCA) cycle, proteomics research helps understand how metabolic pathways work. For example, under certain circumstances, changes in enzyme expression can be a crucial clue to the regulatory mechanism of that pathway [34,86,118,119,120,121]. The relative and absolute expression levels of important enzymes can be analyzed to determine the activation or inhibition of certain metabolic pathways. This information can be used along with metabolic flow analysis to understand how the metabolic pathways are regulated [9,42,122,123,124,125,126,127].

## 4. Central Carbon Metabolism Research and Carbon Source Characteristics

Central carbon metabolism generates the precursor material and energy needed for organisms to grow and survive and underlies major biological processes. Research on the basics of the flux of the intracellular metabolic pathway of *C. glutamicum* was conducted using nuclear magnetic resistance (NMR) spectroscopy in 1995 [126]. In 1996, a study was conducted on the kinetics of the growth rate-dependent modulation through central metabolism and its effect on glucose-limited chemostat cultures [125]. These studies have enabled the quantification of central carbon metabolism, and analyses of the enzyme levels and the growth kinetics of glucose showed that the central metabolism is more complex than previously considered. One study showed that the central metabolism has little in common with general overviews of catabolic pathways, based mainly on gut bacteria. The metabolic pathway remains the same, but the properties and genetic regulation of constituent enzymes suggest that they evolved differently. In 1997, a practical exploration of the pentose phosphate pathway was conducted using NMR spectroscopy. Simultaneously, researchers engineered a *C. glutamicum* L-isoleucine-producing strain, which allowed them to conduct biochemical analyses on intracellular amino acids and flux rates. This study provided a crucial insight, i.e., exporting L-isoleucine was a predominant limiting step in the final reaction [128]. In the 2000s, the central carbon metabolism and 2-methyl citrate cycle investigations were conducted by metabolic profiling using gas chromatography and mass spectrometry (GC-MS), and researchers identified and quantified 74 substances in four metabolic fields (e.g., amino acids, sugars, sugar phosphates, and organic acids) by GC-MS. Gene expressions and metabolite changes in the tricarboxylic acid (TCA) cycle map in acetate-added cultures were studied.

A growth deficiency of a prpD2-mutant strain was observed when propionate was added to cultures growing on acetate, showing that the toxic effect of propionate originated from the accumulation of 2-methyl citrate [119]. In the 2010s, several studies on transcriptional regulators of central carbon metabolism and the genes of enzymes overproduced through fermentation mechanisms under specific conditions were conducted [120,129]. These studies showed that RamA is vital in controlling genes in central carbon metabolism, particularly in the ethanol and acetate metabolism, sugar uptake, glycolysis, the TCA cycle, anaplerosis, and gluconeogenesis. Moreover, RamB plays a major regulatory function in acetate metabolism and acts as a gene repressor during growth on glucose. Studies on the enzyme abundances in *C. glutamicum* were conducted because of the potential in industrial biotechnology [130]. Furthermore, certain enzymatic regulatory patterns depend on the growth conditions, which can be explained partly by the activity of transcriptional regulators [58,120]. In 2017, a comprehensive comparative analysis of *C. glutamicum* sequences through pan-genomic-level analysis techniques with next-generation sequencing (NGS) was conducted, providing conclusive evidence for classifying members of this species. This study provides evidence to accelerate glutamate synthesis in the future [131].

### 4.1. Metabolic Characteristics According to Carbon Source Type

*C. glutamicum* exhibits carbon source-dependent degrees of metabolic flux expression. Basically, the main effect depends on the presence or absence of glucose as determined by carbon catabolite repression (CCR), an environment-sensing mechanism [132,133,134]. In glucose-containing media, metabolic genes are expressed evenly, but under glucose depletion conditions, the genes involved in gluconeogenesis are induced by pyruvic, lactic, acetic, and other organic acids [134]. According to studies on the flux patterns and genome-wide transcriptional profiles of *C. glutamicum*, glycolysis, and the pentose phosphate cycle were strongly induced under glucose-containing conditions. In contrast, the TCA and glyoxylate cycles were highly activated under acetate conditions [119,123,135]. All genes involved in the TCA cycle are co-expressed at the transcriptional level at each growth phase by various substrates, including acetate, glucose, pyruvate, and lactose. In addition, TCA cycle genes are coordinately and specifically regulated by acetate, glucose, or both in the growth medium [136]. Xylose is converted to xylulose by xylose isomerase (encoded by the *xylA* gene), which is phosphorylated to xylulose-5-phosphate by xylose kinase (encoded by the *xylB* gene). After entering the pentose phosphate pathway, it is converted to intermediates, such as ribose-5-phosphate and fructose-6-phosphate, leading to glycolysis and biosynthetic pathways [55,137]. Sorbitol is not used as a carbon source for fermentation, but it is an effective protective agent in increasing the resistance of cells. By co-feeding sorbitol to glucose or adding it to the initial medium, the ability of cells to adapt to environmental changes is promoted. The activity of pyruvate dehydrogenase (PDH), isocitrate dehydrogenase (ICDH), and cytochrome c oxidase (CcO) activities can be maintained at higher levels, and the efficiency of NADH can be improved [138].

In addition, the R2 medium that can use urea as a nitrogen source has been developed, which can benefit cell culture, plasmid amplification, and protein production. Protein production by *C. glutamicum* in BHI media containing pEC-egfp was greater after culture for 12 or 24 h. Because nitrogen metabolism manipulation is closely linked to ammonium assimilation during the synthesis of substances, such as putrescine and L-lysine, it may help to improve these strains further and promote amino acid production through multiple linkages with carbon metabolism. Thus, the utilization of new carbon and nitrogen sources is expected. [52,139,140].

### 4.2. Metabolite Production Based on Carbon Sources

Studies have been conducted on the impact of metabolite production when *C. glutamicum* is grown on carbon sources of glucose, xylose, sucrose, acetate, glucosamine, and molasses. One study claimed L-serine production in high yield using glucose as a carbon source [121]. Serine is not an essential amino acid because it can be synthesized in the human body, but it is involved in the biosynthesis of other amino acids, including glycine, cysteine, and tryptophan. Serine plays an important role in phosphorylation and affects cellular signaling pathways. Furthermore, serine is a critical molecule in the pharmaceutical industry because it is a component of protein and peptide drug synthesis. In addition, serine is a natural moisturizing factor (NMF) and antioxidant used in skincare products and cosmetics. Furthermore, higher glycerate-3-phosphate accumulation was achieved by rewiring the central metabolic pathway, promoting L-serine biosynthesis in high yield [121]. L-leucine is an essential amino acid with various applications, but low productivity inhibits its commercialization. A process with considerable industrial potential has also been devised to produce L-leucine from *C. glutamicum*. This process allows the production of biochemicals derived from intermediates of central carbon metabolism to be scaled up. Increasing the supply of acetyl-CoA and improving glucose uptake was reported to improve L-leucine production [141]. Furthermore, biotechnological efforts have been made to produce β-alanine, 4-amino-3-hydroxybenzoic acid, hydroxybenzoic acids, 5-aminolevulinic acid, and γ-aminobutyric acid using glucose as a carbon source [26,142,143,144,145].

In studies conducted using sucrose as a carbon source for *C. glutamicum*, efforts were made to increase the productivities of L-ornithine, L-serine, and isomaltulose [146,147,148]. Among them, L-ornithine developed *C. glutamicum* strains that can use sucrose or molasses as the carbon source, which can be produced at a lower cost [148]. In addition, L-serine production, a process used widely in the pharmaceutical and cosmetics industries, was improved using sucrose as a carbon source [147]. Isomaltulose is attracting attention as a sucrose substitute because of its unique characteristics, but this process is hindered by uneconomical industrial production. Molasses, an industrial by-product of sugar refining, might be a cost-effective raw material for isomaltulose production by *C. glutamicum*. Moreover, the engineered strains might provide the basis to produce isomaltulose from sugarcane or beet molasses [146].

Anthranilate was biosynthesized using a mixture of glucose and xylose [27]. Anthranilate is chemically produced mainly from petroleum-derived xylene but could be obtained in a biotechnological manner. This compound is an intermediate of the shikimate pathway that can be used as a UV absorber, a perfume that mimics jasmine and orange, and in medicines, such as loop diuretics (e.g., furosemide) [149]. The findings of that study suggested that *C. glutamicum* variants might also be suitable for synthesizing other aromatics. The possibility of producing 1,3-propanediol industrially was also suggested using an approach using glucose and xylose simultaneously [150]. 1,3-Propanediol can manufacture various polyesters, polyethers, and polyurethanes and is used widely as a solvent in the cosmetics industry and a monomer for polymer production. Studies have also shown that *C. glutamicum* can produce 1,5-diaminopentane (cadaverin, a bio-nylon precursor) from xylose. 1,5-Diaminopentane is considered a platform chemical with applications in the automotive industry for producing polymers with enhanced material properties and as a component of biopolyamides. Furthermore, *C. glutamicum* was reported to potentially help produce non-food biomasses, such as lignocellulose, using xylose [55]. In addition, using xylose, methanol, or other basic chemicals to produce non-food regenerative feedstocks using materials like biomasses considered agricultural waste, *C. glutamicum* represents a promising green technology [151].

Glucosamine can also be used to produce amino acids. Glucosamine, an amino sugar abundant in the soil and rhizosphere, can contribute to soil organic nitrogen derived from fungi, invertebrates, and bacterial cell walls. The mutant strain M4 can grow quickly using glucosamine as the only carbon source. The efficient production of L-lysine and diamine putrescine was made possible through proper gene overexpression [152,153,154,155,156]. Table 2 lists the products derived from different carbon sources and presents the supporting literature.

## 5. Conclusions and Prospects

*C. glutamicum* has been studied consistently since its discovery (Figure 1). Physiological studies to use *C. glutamicum* fully have been conducted using various carbon resources on the biosynthesis of various amino acids, aromatic compounds, and natural products (Figure 3). In addition, *C. glutamicum* has been suggested to be a suitable indicator of environmental pollution, a diagnostic tool for pollution, a means of controlled biosynthesis through genetic engineering, and an efficient means of producing drugs and substances useful for food processing. Furthermore, *C. glutamicum* has several advantages over *E. coli* (Table 1), such as product production, tolerance, environmental adaptation, and relatively safe properties. *C. glutamicum* has been considered a promising platform in the biopharmaceutical industry for recombinant protein expression. NMR and GC-MS-based research on the pentose phosphate pathway and central carbon metabolism has elucidated gene expression and metabolite changes during the TCA cycle and revealed the roles of transcriptional regulators. *C. glutamicum* could contribute to the diversification of biomass-derived precursors by biosynthesizing required substances and providing a means of large-scale synthesis. Furthermore, this bacterium can also produce valuable substances using glucose, xylose, and sucrose and can be pretreated to biosynthesize using various carbon sources, such as molasses, glucosamine, and various types of biomasses, if necessary (Table 2). In addition, available data suggest that *C. glutamicum* could synthesize multipurpose compounds, such as isomaltulose, anthranilate, aromatic compounds, and various amino acids. Such processes would be expected to reduce food competition and mitigate environmental issues.

In summary, *C. glutamicum*, a Gram-positive bacterium, is safe for human health by being able to simplify the production process, reduce costs, and facilitate mass production of critical amino acids because it does not have the endotoxin found in Gram-negative bacteria and does not require additional purification steps for endotoxin removal. The advantages of relatively easy genetic manipulation favor optimizing metabolic pathways or introducing new features. *C. glutamicum* can robustly grow under various environmental conditions, making it promising because it has potential applications in industrial production. The flexible central carbon metabolic pathway provides a background for using different substrates.

## Figures and Tables

**Table 1:** Comparative analysis of *Corynebacterium glutamicum* and *Escherichia coli*.

Feature	*C. glutamicum*	*E. coli*
Morphology	Rod-shaped bacterium	Rod-shaped bacterium
Staining properties	Gram (+)	Gram (−)
Genome size	3.28 Mbp	4.6 Mbp
Habitat environment	Soil and plants	Human and animal intestines
Growing environment	Mainly aerobic	Facultative anaerobic
Bacterial endotoxin	X	O
Necessary to form an inclusion body upon recovery	X	O
Expression protein secretion form	High solubility	Low solubility
Plasmid vector optimization	O	O
Protein secretion pathway study	O	O
Safety	GRAS	Most are non-pathogenic *
Industrial application	Amino acid biosynthesis, various compounds biosynthesis, metabolic engineering	Protein production, antibody production, gene cloning, metabolic engineering

O: Possessed characteristic, X: Characteristic not possessed; * Specific pathogenic strains such as *E. coli O157* pose a risk.

**Table 2:** List of metabolites produced from various carbon sources and the corresponding literature.

Carbon Source	Production	Reference
Glucose	L-serine	[139]
	β-alanine	[142]
	4-amino-3-hydroxybenzoic acid	[143]
	5-aminolevulinic acid	[144]
	N-acetylglucosamine	[31]
	Putrescine	[156]
	γ-aminobutyric acid	[26]
	L-cysteine	[76]
	Violacein	[71]
Glucose and xylose	1,3-propanediol	[150]
	Anthranilate	[27]
Xylose	Cadaverin	[55]
Sucrose	L-serine	[147]
	L-ornithine	[148]
Acetate	Itaconic acid	[34]
	Recombinant protein	[97]
Glucosamine	L-lysine, putrescine	[152]
Molasses	Isomaltulose	[146]
	Itaconic acid	[29]
Starch and lignocellulose	Lactate, succinate	[89]

## Data Availability

No new data were created or analyzed in this study. Data sharing is not applicable to this article.
